# South American *Plasmodium falciparum* after the Malaria Eradication Era: Clonal Population Expansion and Survival of the Fittest Hybrids

**DOI:** 10.1371/journal.pone.0023486

**Published:** 2011-09-16

**Authors:** Sean M. Griffing, Tonya Mixson-Hayden, Sankar Sridaran, Md Tauqeer Alam, Andrea M. McCollum, César Cabezas, Wilmer Marquiño Quezada, John W. Barnwell, Alexandre Macedo De Oliveira, Carmen Lucas, Nancy Arrospide, Ananias A. Escalante, David J. Bacon, Venkatachalam Udhayakumar

**Affiliations:** 1 Malaria Branch, Division of Parasitic Diseases and Malaria, Center for Global Health, Centers for Disease Control and Prevention, Atlanta, Georgia, United States of America; 2 Atlanta Research and Education Foundation, Decatur, Georgia, United States of America; 3 Program in Population Biology, Ecology and Evolution, Emory University, Atlanta, Georgia, United States of America; 4 Association of Public Health Laboratories, Silver Spring, Maryland, United States of America; 5 Instituto Nacional de Salud, Lima, Peru; 6 Parasitology Program, Naval Medical Research Center Detachment, Lima, Peru; 7 School of Life Sciences, Arizona State University, Tempe, Arizona, United States of America; University of Missouri-Kansas City, United States of America

## Abstract

Malaria has reemerged in many regions where once it was nearly eliminated. Yet the source of these parasites, the process of repopulation, their population structure, and dynamics are ill defined. Peru was one of malaria eradication's successes, where *Plasmodium falciparum* was nearly eliminated for two decades. It reemerged in the 1990s. In the new era of malaria elimination, Peruvian *P. falciparum* is a model of malaria reinvasion. We investigated its population structure and drug resistance profiles. We hypothesized that only populations adapted to local ecological niches could expand and repopulate and originated as vestigial populations or recent introductions. We investigated the genetic structure (using microsatellites) and drug resistant genotypes of 220 parasites collected from patients immediately after peak epidemic expansion (1999–2000) from seven sites across the country. The majority of parasites could be grouped into five clonal lineages by networks and AMOVA. The distribution of clonal lineages and their drug sensitivity profiles suggested geographic structure. In 2001, artesunate combination therapy was introduced in Peru. We tested 62 parasites collected in 2006–2007 for changes in genetic structure. Clonal lineages had recombined under selection for the fittest parasites. Our findings illustrate that local adaptations in the post-eradication era have contributed to clonal lineage expansion. Within the shifting confluence of drug policy and malaria incidence, populations continue to evolve through genetic outcrossing influenced by antimalarial selection pressure. Understanding the population substructure of *P. falciparum* has implications for vaccine, drug, and epidemiologic studies, including monitoring malaria during and after the elimination phase.

## Introduction

Understanding the population dynamics of malaria parasites can provide useful insights for malaria elimination programs [1]. The international malaria eradication campaign initiated during the early 20^th^ century resulted in the elimination of malaria in some regions of the world [2], [3]. Despite these efforts, malaria reemerged largely due to the failure of traditional control programs. It is not well understood how the malaria eradication campaign altered the population structure of malaria parasites, but it likely led to population bottlenecks.

In South America, earlier studies of *P. falciparum* population structure provided evidence for clonal parasite populations due in part to bottlenecks caused by control, as well as low transmission (<1%; [4]), inbreeding, and epidemic expansions. Populations have limited genetic diversity, high differentiation, and are out of mutation drift equilibrium (MDE) [4], [5], [6], [7], [8], [9], [10], [11], [12], [13], [14], [15]. When populations are in MDE, the number of mutations entering the population is balanced by the number of mutations being removed by random change, called genetic drift. The disruption of MDE indicates the effective population has not been stable over time. Population differentiation occurred through fragmentation, genetic drift in small effective population, the creation of new populations from migrants (founder effect), and subsequent admixture between sites [4]. Tibayrenc and Ayala have postulated that *P. falciparum* propagation may be essentially clonal in such situations [16], [17]. They suggested such lineages be referred to as clonets, which are genetically identical for a set of markers, but potentially variable at others, with the most recent common ancestors occurring weeks to hundreds of years ago [16], [17].

In this study we investigated the consequences of malaria eradication and drug pressure on the genetic structure of *P. falciparum* populations in Peru. Control efforts achieved near elimination of malaria from 1966 to 1989 [18]. This ended when Peru suffered multiple epidemics of malaria during the 1990s [19], reaching peak incidence in 1998 on the Pacific Coast and in the Amazon [1], [20]. Subsequent control reduced malaria throughout Peru, particularly on the Pacific Coast. Yet it continues to be transmitted in the Amazon, with the majority of cases in the department of Loreto, especially in Iquitos, the largest city in the region [21]. Due to the resurgence of malaria, clinical trials were conducted in 1998–2000 and showed that strains from the coast and the western Amazon were chloroquine (CQ) resistant, but sulfadoxine pyrimethamine (SP) sensitive, while strains from the remainder of the Peruvian Amazon were CQ and SP resistant [1], [22]. One study suggested there were two parasite lineages in Loreto based on *pfcrt* alleles (SVMNT and CVMNT) [23]. Another suggested there were three lineages based on clinical resistance (a Brazilian, a Loreto, and a Western Amazon/Pacific type) [21].

As a consequence of these studies, in 2001 Peru adopted artesunate plus mefloquine combination therapy (ACT) for the Amazon region and artesunate plus SP for the coastal region as the primary *P. falciparum* treatments [1]. Thereafter, the frequency of SP resistant alleles of *dhfr* and *dhps* significantly decreased in the Amazon, possibly due to the higher fitness of sensitive alleles. An earlier shift from CQ to SP in 1995 did not lead to a reduction in CQ resistant alleles [24], [25] because the essential K76T mutation in *pfcrt* was fixed [26], [27].

These historical events, combined with the availability of parasite specimens from studies conducted immediately after the peak of malaria resurgence (1999–2000) and several years after the introduction of ACT (2006–2007), allowed us to test three hypotheses: 1) that the resurgence of *P. falciparum* after the eradication era may have been due to a) clonal expansion of residual parasites or b) expansion of recently introduced parasites from elsewhere in South America; 2) that regional differences in drug resistance profiles may suggest underlying population structure with the Andes Mountains acting as a barrier to gene flow; 3) that changes in drug policy a) only altered the frequency of resistant alleles and did not influence the frequency of alleles elsewhere in the genome during the period of increased malaria transmission or b) influenced both due to limited opportunities for sexual recombination.

## Materials and Methods

### Ethics Statement

The parasite samples used in this study were obtained from sample collection protocols that were approved by the Ethical Review Committees of the Instituto Nacional de Salud (for northern Pacific Coast), US Naval Medical Research Center Institutional Review Board and the National Institutes of Health of Peru (for Peruvian Amazon); and Institutional Review Boards of the U.S. Army, the U.S. Navy, and the Universidad Cayetano Heredia (for central and northeastern Amazon) [20], [22], [24], [25], [26], [28] as well as the U.S. Centers for Disease Control and Prevention. Written informed consent was provided by study participants and/or their legal guardians. Samples obtained from Iquitos was approved by the U.S. Naval Medical Research Center Institutional Review Board (approval no. NMRCD.2000.0006).

### Study Sites and *P. falciparum* Clinical Isolates

We examined 220 Peruvian *P. falciparum* clinical isolates collected during 1999–2000 from the northern Pacific Coast (Bellavista, n = 2; La Arena, n = 11 and Zarumilla, n = 67), the western Peruvian Amazon (Pampa Hermosa, n = 10; Ullpayacu, n = 25) collected during drug efficacy trials [20], [22], [25], and from the central Peruvian Amazon (Padre Cocha, n = 65) and the eastern Peruvian Amazon (Caballococha, n = 40) collected during drug efficacy trials and surveillance studies [24], [26], [28].

We also examined 62 Peruvian *P. falciparum* clinical isolates collected during 2006–2007 in Iquitos during an ongoing febrile surveillance study. We used the Padre Cocha isolates as a historical comparative time point for Iquitos.

### DNA Isolation, PCR Amplification and Genotyping of dhfr, dhps, pfcrt, and pfmdr1

DNA was isolated from filter paper blood spots [22], [25], [26] or whole blood [26] using the QIAamp DNA blood mini kit (QIAGEN, Valencia, CA). Samples from Padre Cocha, Caballococha, and Iquitos were previously sequenced for point mutations in *dhfr*, *dhps*, *pfcrt*, and *pfmdr1* [26]. Limited samples from these sites, as well as from the Iquitos, were resequenced for *dhps* to test for a novel synonymous *dhps* mutation at codon 540 (AAG). Sequencing of *dhfr* and *dhps* in Pampa Hermosa and Ullpayacu samples were previously reported [22] and were resequenced for confirmation. Samples from Bellavista, La Arena, Pampa Hermosa, Ullpayacu, and Zarumilla were sequenced for *pfcrt*, *pfmdr1*, and *dhps* using protocols described previously [9], [29]. A nested PCR protocol was utilized for *dhfr*, with outer forward primer 5′-TCCTTTTTATGATGGAACAAG-3′ and outer reverse primer 5′-AGTATATACATCGCTAACAGA-3′. The secondary reaction, utilized 5′-TTTATGATGGAACAAGTCTGC (forward) and 5′-ACTCATTTTCATTTATTTCTGG-3′ (reverse) primers. The cycling conditions were 94°C/5 min; 35 cycles of 95/30 sec, 50/30 sec, 68/1 min; 68°C/5 min (primary reaction) and 94°C/5 min; 30 cycles of 95°C/30 sec, 52°C/30 sec, 68°C/1 min; 68°C/5 min (secondary reaction).

### Microsatellite Typing

Whole genome amplified DNA (Qiagen's REPLI-g Whole Genome Amplification Kit, Valencia, CA) was used for microsatellite characterization. Samples were assayed for 12 microsatellite loci spanning 499.5 kb around *pfcrt* on chromosome 7; 15 microsatellite loci spanning 544.7 kb around *pfmdr*1 on chromosome 5; 13 microsatellite loci spanning 700 kb around *dhfr* on chromosome 4; and 16 microsatellite loci spanning 406.3 kb around *dhps* on chromosome 8 [30], [31], [32]. Primer sequences and their PCR parameters were described earlier [9], [11]. We previously reported microsatellite data close to *dhfr*, *dhps*, *pfcrt*, and *pfmdr1* for Caballococha and Padre Cocha [26]. In an earlier paper, we created haplotype identifiers for each of these genes based on microsatellite loci that were nearby [26] These haplotypes are denoted by suffixes after gene alleles and their lettering scheme is not related to the identifiers used for clonets. In addition, we examined 12 putatively neutral microsatellite loci. Five were selected from neutral markers previously described (TA1, chromosome 6; poly α, ch. 4; PfPK2, Ch. 12; TA109, ch. 6; and 2490, ch. 10) [4], [33]. The remaining seven markers were C2M33, C2M34, C2M29, C2M27 on ch. 2; and C3M40, C3M69, and C3M39 on ch. 3 [11].

### Statistical Analysis

We used seven microsatellites on different chromosomes (TA1, poly α, PfPK2, TA109, 2490, C2M34, and C3M69) to examine Peruvian *P. falciparum* population structure between 1999–2000. A locus-by-locus hierarchical analysis of molecular variance (AMOVA) was used to partition variation among and between all populations, as well as between coastal and all Amazonian sites using Arlequin version 3.1 [34]. Significance of the fixation indices was determined using a non-parametric approach. F_ST_ was calculated among all populations, with the exception of Bellavista because it was represented by only two samples, and between all pairs of populations. The significance of F statistics and genetic variance components were tested using 1,000 permutations [34]. We also used F_ST_ to compare clonal lineages.

Isolation by distance was tested by regressing pairwise F_ST_ on pairwise geographic distances among populations [35] and significance determined with Mantel's tests (1,000 permutations) using Arlequin [34]. We tested whether grouping our samples by apparent ancestral populations explained more genetic variation than grouping them by collection sites using AMOVA. We initially examined the seven neutral markers and then expanded to all microsatellite markers including those considered neutral and flanking drug resistance genes, with the exception of *dhfr*: 0.52 kb and *dhps*: 9.0 kb.

Significant associations between microsatellite loci within clonets were determined using an exact test of linkage disequilibrium (LD) [36] and 10,000 Monte Carlo steps in Arlequin version 3.1 [34] and a Bonferronni-Holms correction [37].

We tested for bottlenecks using Bottleneck (www.ensam.inra.fr), which assumes that utilized markers are neutral and not in LD, and that populations lack substructure, migration, and hybrids. When a population is at MDE, each microsatellite should have an equal probability of having an observed H_e_ deficit or excess in comparison to the expected H_e_ based on the number of alleles. After a bottleneck, there will be a reduction in the number of alleles and H_e_ at polymorphic loci. However, allelic diversity decreases at a faster rate than H_e_ during a bottleneck. Therefore, a bottleneck is indicated if a significant number of loci have a H_e_ excess compared to that expected if the population was in mutation-drift equilibrium. Conversely, if there is H_e_ deficit, the population will also no longer be in MDE and a rapid population expansion is indicated. To test whether our populations were in MDE, we used a sign test, which assumes a null hypothesis of MDE, but has low statistical power. To test for H_e_ deficits and excesses, we used a Wilcoxon sign-rank test [38]. We used a two-phased model of mutation for all tests [38] and included the seven neutral markers, as well as four markers from each chromosome carrying one of the genes (Ch. 4, 347.1 kb; Ch. 5, −305 kb; Ch. 7, −257 kb; and Ch. 8, −196.6) selected to be as far from the genes as possible.

### Network Analysis

We created two median-joining network diagrams using Network v. 4.516 (fluxus-engineering.com) [39]. First, we used the seven microsatellites previously described to examine samples collected between 1999–2000 for clonal structure, but did not include the additional four markers in order to maximize the sample size; there were samples, particularly those collected in the Western Amazon, which we would have had to exclude if we had included all eleven markers. Second, we expanded to the eleven microsatellites for another network diagram to illustrate the genetic relationships between isolates collected in Iquitos and isolates from 1999–2000, though this reduced the number of usable samples. Additional markers were included in the second network diagram to increase the resolution of our network diagram and to demonstrate the robustness of our findings.

## Results

### Analysis of Population Structure in 1999–2000 Reveals Clonal *P. falciparum* Lineages

We analyzed *P. falciparum* isolates from sites across Peru collected during 1999–2000 to understand underlying population structure. For F_ST_ analysis, seven neutral microsatellite markers from different chromosomes were used. Statistically significant pairwise F_ST_ ranged from 0.22 (Padre Cocha and Pampa Hermosa; Padre Cocha to Caballococha) to 0.9 (Zarumilla and Ullpayacu), which suggests the parasites from various sites are differentiated (Table 1). Padre Cocha is most similar to Caballococha and Pampa Hermosa, and more differentiated from La Arena, Ullpayacu, and Zarumilla. The significant differentiation between Zarumilla and La Arena (F_ST_ = 0.58) may be due to the limited sampling of La Arena (n = 11), as they had the same circulating neutral alleles. Ullpayacu is highly differentiated from all the other sites. Pampa Hermosa is most similar to La Arena and Padre Cocha. No isolation by distance was found in Peru based on the Mantel test (R^2^ = 0.01, p = 0.447).

**Table 1:** Pairwise F_ST_ by Collection Site or Clonet.

Study Sites	n*	Zarumilla	La Arena	Padre Cocha	Caballococha	Pampa Hermosa
Zarumilla	66	-	-	-	-	-
La Arena	11	0.58	-	-	-	-
Padre Cocha	58	0.61	0.40	-	-	-
Caballococha	38	0.62	0.39	0.22	-	-
Pampa Hermosa	7	0.48	0.28	0.22	0.31	-
Ullpayacu	24	0.90	0.85	0.52	0.55	0.77

Clonet	n	A	B	C	D	E
A	30	-	-	-	-	-
B	22	0.80	-	-	-	-
C	33	0.80	0.83	-	-	-
D	42	0.83	0.85	0.70	-	-
E	81	0.86	0.84	0.89	0.78	-

Pairwise F_ST_ values calculated when comparing different collection sites or clonets using the 7 neutral markers described in the text. All values are significantly different from zero (p≤0.05).

*n denote sample size.

**Figure 1:**
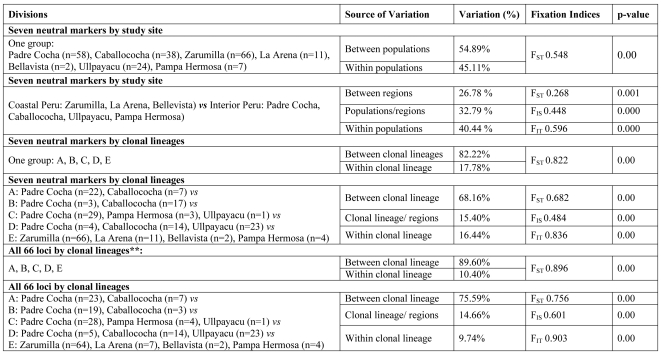
AMOVA Results. Locus by locus AMOVAs were used to create this table. N denotes sample size. Haplotype group refers to a combination haplotype of the haplotypes seen around each of the four genes. ** Two apparently monomorphic markers were removed from analysis (*dhfr*: 0.52 kb and *dhps*: 9.0 kb) due to poor amplification in clonet D.

The lack of spatial population structure, and prior evidence suggesting clonality in South America, led us to examine population structure based on grouping parasites into clonal lineages. We identified five clonal lineages in multilocus linkage disequilibrium (LD) using the same seven neutral satellite markers. Each clonal lineage carried one allele at each locus in 80–100% of isolates and we designated them clonets A, B, C, D, and E (Table 2). Statistically significant F_ST_ values between the clonets exceeded 0.70 in all cases, suggesting high differentiation between them (Table 1). We also illustrate that each clonet has few polymorphic markers and levels of pairwise LD for the remaining polymorphic that are higher than the expectation of random chance (p = 0.05; Table 2). Applying AMOVA to these markers, only 27% of variation was explained by comparing sampling sites while 68% of variation was explained by clonets (Fig. 1). Furthermore, when the AMOVA was expanded to all 66 markers, partitioning the data by site only explained 7% of the variation (data not shown), while clonets explained 76% of variation. The clonality is also illustrated by the few polymorphic markers in each clonet and high levels of pairwise LD for the remaining polymorphic markers (Table 2). All subsequent analyses defined populations based on clonets because it maximized population differentiation.

**Table 2:** Most common alleles in clonets.

Clonet	TA1,^1^, Ch. 5^2^	poly α, Ch. 4	PfPK2, Ch. 7	TA109, Ch. 6	2490, Ch. 10	C2M34, Ch. 2	C3M69, Ch. 3	Monomorphic markers, 66 markers^4^	Pairwise LD in polymorphic markers
A	169; 84%^3^	172; 100%	166; 93%	164; 100%	84; 93%	240; 100%	132; 55%	70%; n = 29	11%
B	172; 90%	183; 100%	172; 100%	164; 85%	84; 100%	226; 100%	149; 100%	58%; n = 23	12%
C	178; 56%	164; 97%	163; 97%	160; 94%	80; 85%	246; 85%	136; 85%	40%; n = 33	30%
D	178; 56%	161; 80%	175; 82%	160; 100%	80; 98%	233; 100%	122; 100%	44%; n = 39*	31%
E	172; 99%	148; 99%	175; 84%	160; 99%	74; 83%	226; 100%	138; 83%	83%; n = 84	21%

Values represent most common fragment sizes in nucleotides, followed by the percentage of isolates carrying them.

*Two apparently monomorphic markers were removed for analysis of clonet D due to poor amplification (*dhfr*: 0.52 kb and *dhps*: 9.0 kb).

1 The microsatellite loci name.

2 its chromosome,

3 the first value indicates the common allele size and the second is the percentage of isolates carrying it.

4 The column represents the number of monomorphic markers out of the total 66 markers examined in this study as a percentage, along with the number of samples used to calculate this value.

### Genetic Relatedness between Clonets and Their Geographic Distribution

**Figure 2:**
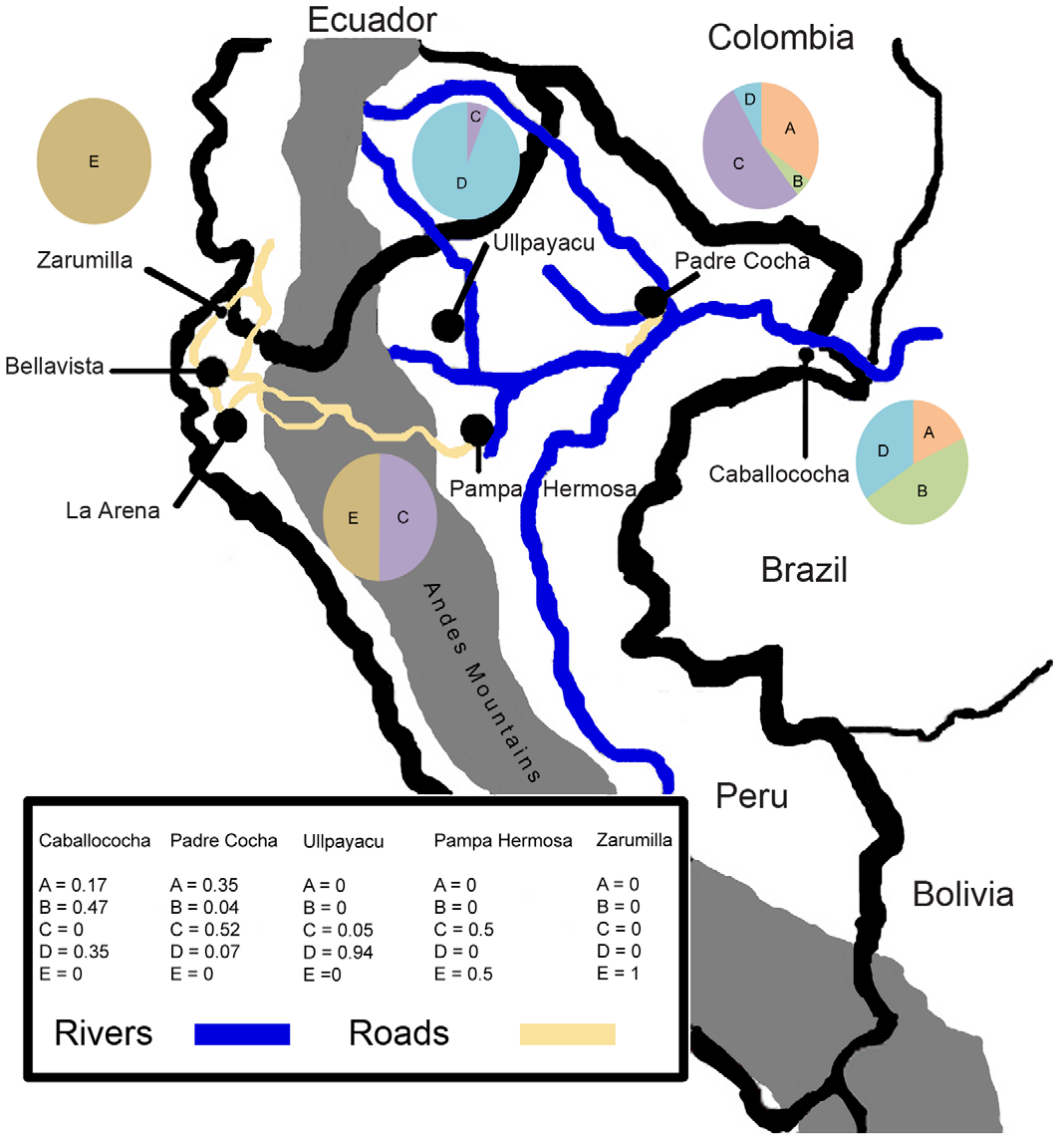
Sample Collection Sites and Distribution of Clonets. A map of Peru that shows the collection sites, as well as the Andes Mountains (dark gray), roads of interest (black lines over Andes), and rivers of interest in the Amazon (light gray). Iquitos is omitted from the map. It is located in the black dot representing Padre Cocha. Roads and rivers are drawn in yellow and blue respectively. They are not meant to represent all of the roads in Peru and are truncated in Ecuador. They were included in order to suggest how parasites could have migrated within Peru and been introduced through Ecuador. The proportion of clonets in study sites is indicated in the map legend and pie charts.

**Figure 3:**
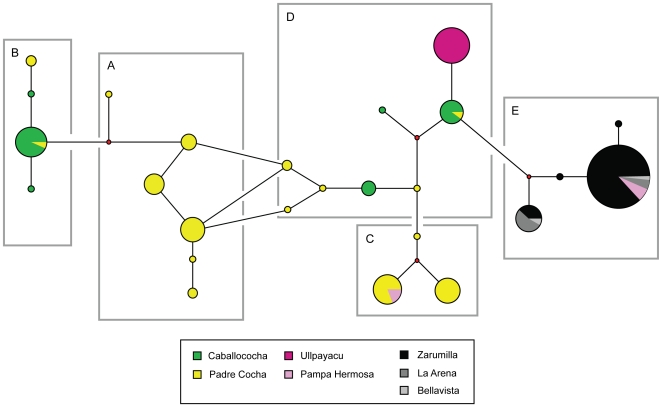
Network Analysis of Clonets Integrated with Study Sites. Network diagram showing the genetic relationships among the A, B, C, D, and E clonets and collection sites from 1999–2000 using the seven neutral microsatellite markers described in the text. Small red circles represent hypothetical nodes that link haplotypes seen among our samples.

The distribution of different clonets and their relative proportions at each site is indicated in figure 2. The Pacific coast sites (Bellavista, La Arena, and Zarumilla) only had the E clonet. Ullpayacu, a western Amazon site, had only the D clonet, with the exception of one sample from the C clonet. Despite limited isolates from Pampa Hermosa, both the C and E clonets were found. In Padre Cocha, clonets A, B, C, and D were found, while in the Caballococha the A, B, and D clonets were found. The geographic distribution of these clonets suggested that the highest amount of admixture could be found in Padre Cocha, the site closest to Iquitos. A median joining network analysis was performed to understand the genetic relatedness between the clonets (Fig. 3). This analysis indicated clonet B is only linked to clonet A, clonet C is only linked to clonet D, and clonet E was only linked to clonet D, which suggested the potential shared ancestry of these pairings. However, Clonet D was found at many sites and linked to clonets A, C, and D, suggesting it could have sexual recombined with some of them.

### Evidence for Clonet Bottlenecks or Population Expansions

Testing for bottlenecks requires polymorphic markers which are inherently difficult to locate in clonets. Overall, clonets A, B, and E had more monomorphic markers than clonets C and D. Clonet E has the least microsatellite variation. The sign test did not reject MDE for any of the clonets, though it was almost rejected for clonet B (p = 0.07). According to the Wilcoxin sign-rank test, only clonet E had a significant H_e_ excess (p = 0.03), suggesting a recent bottleneck. Only clonet B had a significant H_e_ deficit (p = 0.03), indicative of a rapid expansion.

### Drug Resistance Associated Alleles Correlate with Clonets

**Figure 4:**
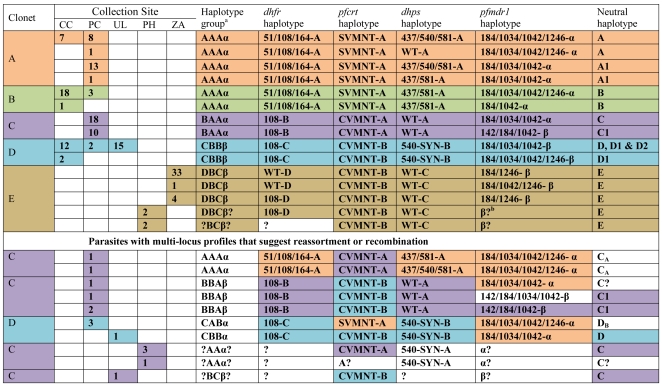
Drug Resistance Multilocus Linkage Disequilibrium. The multiallelic disequilibrium seen across multiple genes and microsatellite markers. **^a^**Haplotype groups are summaries of the haplotypes found for the following four columns. ^b^“?” means that there was incomplete or unknown data for this cell. The final column suggests the subvariants seen within each clonet amongst the seven neutral markers. Notice that clonet C was found with both *pfmdr1* lineages; while we would argue the α is ancestral, there is no direct proof of this. In cases were reassortment or recombination appears to have occurred, we have suggested the secondary clonet with a subscript (e.g. C_A_ was most likely a cross between clonet A and C.) SYN is an abbreviation for synonymous. Each clonet is identified by a unique color and the color coding is also used in the figures.

We hypothesized that the distribution of clonets at collection sites might be associated with the drug resistance profiles seen in different regions (Fig. 4). To test for drug resistance genotypes, we amplified and sequenced four genes (*pfcrt*, *pfmdr1*, *dhfr*, and *dhps*) previously implicated in resistance. Triple mutant *dhfr* alleles were found almost exclusively in clonets A and B, with the exception of two isolates belonging to clonet C (potential recombinants). Triple mutant *dhps* alleles were exclusively found in clonet A, while clonet B had only the double mutant *dhps* allele, except the same two potential recombinants as above. Clonets A and B carried CQ resistant *pfcrt* allele SVMNT-A. Clonet C carried CVMNT-A, an allele that may be ancestral to SVMNT-A. Clonet C also carried a single mutant *dhfr* allele (108N-B) in most cases, along with the wild type *dhps*-A allele, which may have been ancestral to the alleles found in clonet A and B. Clonets D and E carried SP sensitive alleles. Clonets D and E carried a CVMNT-B allele not shared with clonet A, B, or C. Clonet D carried a 108-C *dhfr* allele and a unique *dhps* wild type allele with a synonymous mutation at codon 540. Clonet E carried a *dhfr* wild type allele or a mutant 108-D. It also carried a *dhps* wild type-C allele. Two major *pfmdr1* lineages (α and β) were found. These were based on the proximal −1.40 and 0.45 kb microsatellite markers [26]. The α lineage had a 197 bp in length allele and a 191 bp in length allele at the two respective loci, while the β lineage had a 203 bp and a 178 bp alleles. The α lineage was predominately seen in the A, B, and C clonets, along with a few samples from the D clonets. The β *pfmdr1* lineage was only seen in the C, D, E clonets. The multilocus LD seen between the 66 microsatellite markers extended to *pfcrt*, *pfmdr1*, *dhfr*, and *dhps* alleles. However, the multilocus lineages had partially broken down in a few isolates carrying clonet C and D, particularly those isolated in Padre Cocha. This suggests there may have been some reassortment and recombination.

### Clonet Breakdown and Selection Due to Increased Transmission, Diversity, and Drug Pressure

**Figure 5:**
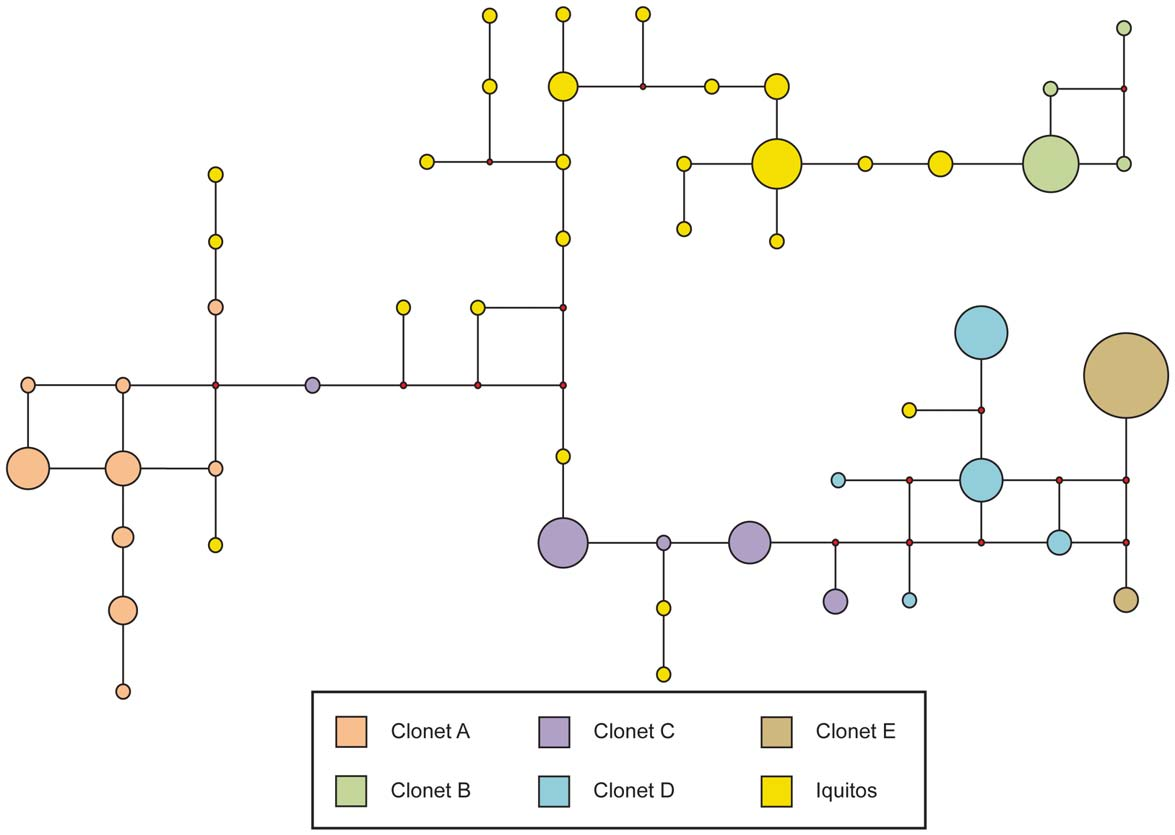
Network Analysis of Clonets in 1999 and Relatedness to Iquitos Clonets in 2006–2007. A network diagram of Iquitos in comparison to clonets calculated from neutral marker data from 1999–2000. This network diagram shows the genetic relationships between Iquitos and the previously reported clonets A, B, C, D, and E using the eleven neutral microsatellite markers described in the text. Small red circles represent hypothetical nodes that link haplotypes seen among our samples.

**Figure 6:**
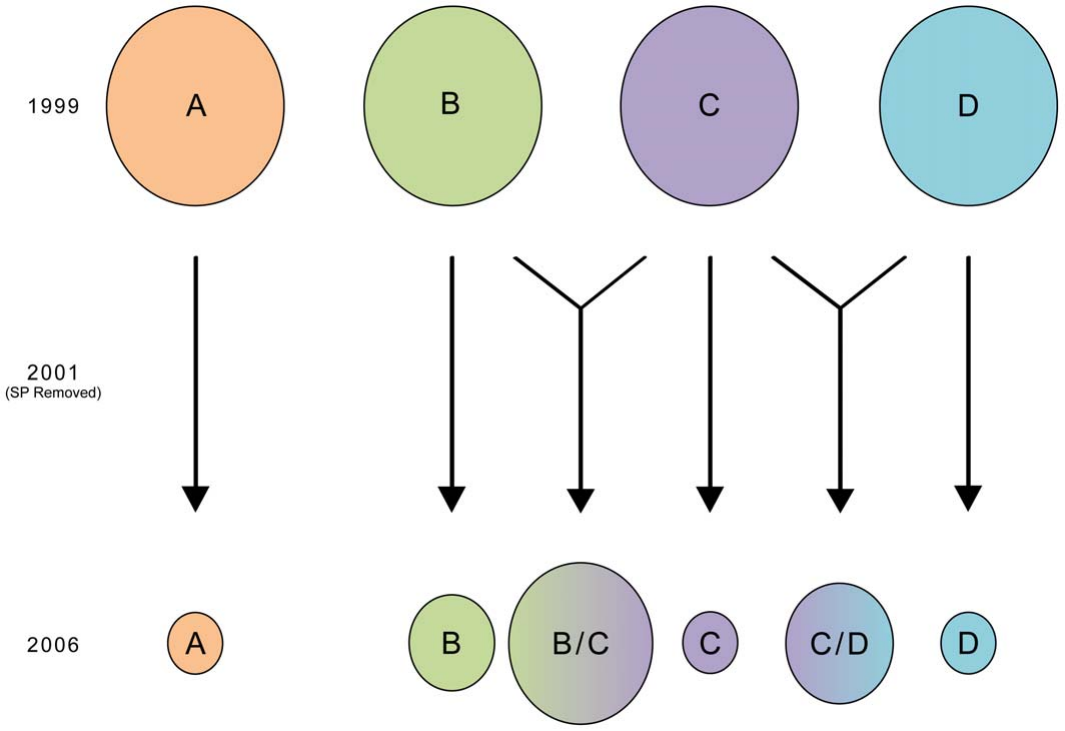
Schematic of Changes in Clonet Profiles between 1999 to 2007. Each circle represents a different clonet. After the removal of SP, the four clonets seen in Padre Cocha have been reduced in size in favor of a number of hybrids. The most common of these represent hybridizations of clonet B and C or C and D. Note, however, that the lack or low levels of other simple crosses of the various clonets, which suggests selection by drug pressure may have influenced surviving offspring.

To test whether changes in the drug policy has influenced population dynamics, we compared the 1999 clonet profile and resistant lineages with the 2006/7 data from Iquitos (Table 3 and Fig. 5). With no interbreeding, the clonet structure in Iquitos should have been maintained (persistence of clonets A, B, C, and D). If there had been panmixia, we would have expected hybridization between at least some of these clonets (Fig. 6 and Table 3). Isolates from 2006–07 often clustered independently from clonets A, B, C, D and E in a median joining network (Fig. 5), though some clustered with clonet B, and a very few with clonets A,C, and D. A large number of isolates were hybrids of clonet B and C or C and D (Table 3). Additional recombination between clonets A, B, and C, or B, C, and D is suggested by the few remaining samples. As clonet A was in high frequency in 1999 in Padre Cocha, the scarcity of clonet A hybrids in 2006–07 was surprising. On the other hand, hybrids of clonet C and B were at higher frequency than expected through random mating. Taken together, this suggests that there was selection for clonet hybrids that carried SP sensitive genotypes (Table 3 and Fig. 5). While pairwise LD appeared similar to that seen in 1999–2000, the number of monomorphic markers decreased, as only 8 of the 66 markers were monomorphic (n = 32) though 28% of comparisons were in significant linkage.

**Table 3:** Drug resistance allele haplotypes seen in Iquitos in 2006–2007.

n*	Haplotype group	*dhfr*	*pfcrt*	*dhps*	*pfmdr1*
1	AAAα	51/108/164-A	SVMNT-A	437/540/581-A	α-184/1034/1042
6^i^	AAAα	51/108/164-A	SVMNT-A	437/581-A	α-184/1034/1042/1246
1	AAAβ	51/108/164-A	SVMNT-A	437/581-A	β-142/184/1042
3	DAAα	50/51/108-D	SVMNT-A	437/540/581-A	α-184/1034/1042/1246
1	AAAβ	51/108/164-A	SVMNT-A	WT-A	β-142/184/1042
1	ABAβ	51/108/164-A	CVMNT-B	WT-A	β-142/184/1042
17^ii^	BAAα	108-B	SVMNT-A	WT-A	α-184/1034/1042
5	BAAα	108-B	SVMNT-A	WT-A	α-184/1034/1042
1	BAAβ	108-B	SVMNT-A	WT-A	β-142/184/1034/1042
2	BAAβ	108-B	SVMNT-A	WT-A	β-142/184/1042
1	BAAβ	108-B	SVMNT-A	WT-A	β-184/1042
3iii	BAAα	108-B	CVMNT-A	WT-A	α-184/1034/1057
12^iv^	BBAβ	108-B	CVMNT-B	WT-A	β-142/184/1048
1	BBAβ	108-B	CVMNT-B	WT-A	β-142/184/1034/1042
1	?BAβ	?	CVMNT-B	WT-A	β-142/184/1049
2	CBCβ	108-C	CVMNT-B	WT-C	β-184/1034/1042
2^v^	CBBβ	108-C	CVMNT-B	WT-AAG-B	β-184/1034/1042

The multiallelic linkage disequilibrium between drug-resistance alleles. It does not include neutral haplotypes because of the extent of chromosomal reassortment.

i 5/6 of these samples represented clonet B.

ii 16/17 of these samples appear to represent the same combination of clonet B and C.

iii 2/3 of these samples represented clonet C.

iv 7/12 of these samples represented the same combination of clonet C and D.

v These samples represented a combination of clonet C and D.

*n denotes sample size. Haplotype group refers to a combination haplotype of the haplotypes seen around each of the four genes.

## Discussion

This study indicates that Peruvian *P. falciparum* populations expanded from bottlenecked populations or migrants in the post-eradication era. During the peak malaria incidence (1998–2000), Peruvian parasite populations consisted of at least five clonal lineages with varying drug resistant genetic backgrounds. Since the 1990s the increase in the transmission intensity has favored sexual recombination, especially in the Central Amazon region. Simultaneously, changes in drug policy seem to have been a critical selective force. We argue that the combination of increased opportunities for outcrossing and changes in drug policy strongly influenced population structure and parasite evolution between the study time points.

Previous studies have suggested that low transmission could maintain linkage disequilibrium in *P. falciparum* [4], [6], [9], [10], [11], [12], [40], [41]. However, the mechanisms underlying population differentiation in low transmission areas have not been explained, beyond invoking genetic drift [4], [7], [42] or, rarely admixture [4]. Our study clearly demonstrates how these factors have influenced parasite populations. We demonstrate how clonet expansion and migration can alter parasite population structure in a region after the era of eradication. We show that multiple clonets can be maintained in sympatry if there is not sufficient transmission for outcrossing.

Historical malaria eradication may have reduced parasite populations to a patchy network of refuges in areas of low *P. falciparum* transmission like South America [3], [43]. These refuges could be interpreted as the kind of islands that Sewall Wright addressed with his island model of population structure [41]. In the island model, the population is subdivided into subgroups with each breeding within itself, except for migrants [44]. These subgroups can be due to geography, ecology, or time [45]. In the case of South America, malaria eradication would have led to separated, allopatric parasite refuges. These, in turn, would have eventually led to clonal lineages developing through genetic drift and inbreeding. After malaria eradication efforts waned, such populations would have expanded locally and begun to migrate to other islands of inbred parasites. The opportunities for outcrossing between these coexisting, sympatric, clonets would have been limited because of the low frequency of multiple infections in a low transmission environment.

The demonstration that Peru had at least five clonal lineages of *P. falciparum* in the post eradication era is consistent with other recent studies around Iquitos [46], [47]. In one, whole genome sequences of 14 *P. falciparum* isolates led to the conclusion that the isolates were largely identical, with at most four parental haplotypes [47]. Another argued that five independent clusters of related subpopulations exist based on microsatellite data collected between 2003–2007 [46]. Our study was able to draw more general conclusions because it utilized samples from across the country. Furthermore, many samples were collected around 1999, after the peak of the malaria resurgence, which suggests that only a few lineages were involved.

Genetic drift caused by self-mating, mitotic replication, or other processes is an important source of genetic diversity in areas of clonal propagation like South America. Over the short term, however, clonets might have been maintained if there was only one clonet present in each locality, as on the Pacific coast of Peru, or had there been insufficient transmission for outcrossing to occur among clonets. We argue that the increase in malaria transmission in the late 1990s led to admixture and cryptic parasite population substructure (Fig. 4). Furthermore, we argue that the rapid increase in malaria transmission during the late 1990s led to sufficient multiple infections for clonet outcrossing to occur and that this was a greater influence on genetic diversity than the genetic drift that likely generated the clonets. Another recent study has confirmed the frequent occurrence of recombination in this region as 33.6% of infections examined in a cohort study involving villages near Iquitos between 2003–2007 were mixed, with a decrease in LD over time, and recombination in later years [46].

Clonal substructure was found at all of the collection sites in the Amazon interior (clonets A, B, C, D, and E) and a single clonet expansion was found on the northern Pacific coast (clonet E). The Andes appeared to act as a semi-permeable barrier to gene flow, but, within the Amazon interior no barrier was found. Though the coastal (clonet E) and western Amazon sites (C and E vs. D and C) do not share many clonets, Caballococha (A, B, C) and Padre Cocha (A, B, C, D) shared three. The presence of a few clonet E isolates in Pampa Hermosa suggests a recent introduction, perhaps in the early 1990s, to the Western Amazon by way of Andean roads that terminate near Pampa Hermosa [48]. The comparative genetic diversity seen in Padre Cocha is not surprising given its close proximity to Iquitos, where ∼42% of Loreto's population lives [21]. Iquitos has a large enough population to support multiple lineages, is a hub of human movement, and many *P. falciparum* cases were reported there, allowing for potential multiple infections and recombination.

Historical facts about the origins of CQ and SP resistance in different time points allow us to speculate when different clonets may have migrated to Peru. The first reports of CQ resistance in Peru occurred in the eastern Amazon in 1979–1980, while parasites to the south remained CQ-sensitive [48], [49]. Peruvian reports occurred decades after the first continental reports from Venezuela (1959; [50]) or Rondônia, Brazil (1960; [50]). It was suggested that resistance may have spread from coastal and interior Ecuador, where it was reported as early as 1976 [21], [50], [51]. However, nearby Rondônia, Brazil might have also been a source of Peruvian CQ-resistant parasites. Molecular data suggested at least two independent origins for CQ resistance in South America [32], which we identified as the *pfcrt* CVMNT-B allele (coastal and western regions of South America) and the CVMNT-A/SVMNT-A alleles (Amazon). Our data indicates that these two CQ resistant lineages have colonized Peru with the CVMNT-A/SVMNT-A alleles mostly occurring in the eastern and central Amazon region and the CVMNT-B allele on the Pacific coast and in several sites in the Amazon [32]. SP resistance was proposed to have developed in the Amazon as early as the 1970s and spread regionally [7], [52], but was not reported in Peru until the 1990s.

**Figure 7:**
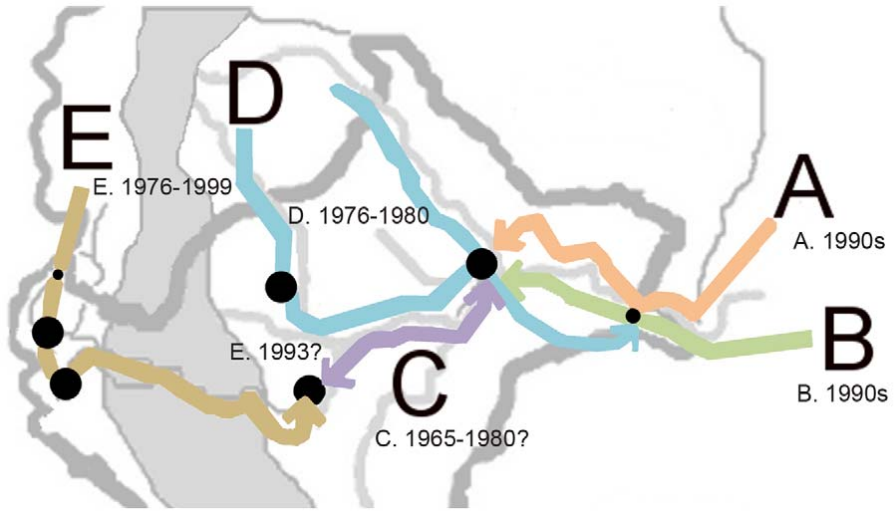
Hypothesized Spread of Clonets Across Peru. Clonet A: orange, B: green, C: purple, D: blue, E: brown. Our hypothesis of how these clonets may have spread through Peru is described in the text. In brief, we suggest that clonets A and B may have been recently introduced from the greater Amazon Basin, that clonet C may represent an older vestigial population, that clonet D may have been introduced during the 1980s from theAmazon interior of Ecuador, and that clonet E may represent a coastal lineage that has recently invaded the interior of Peru.

We were able to hypothesize when the five clonets were introduced into Peru based on their drug resistance profiles. Clonets A and B always had the S_tct_VMNT *pfcrt* allele associated with highly resistant *dhfr* and *dhps* alleles (noted in the Amazon by others as well [7]), along with the *pfmdr1* α lineage. Therefore, it appears clonet A and B may have swept through the Amazon basin from Brazil and expanded during the late 1990s when SP was introduced for primary treatment of malaria in Peru (Figure 7). The absence of the A and B clonets in the western Peruvian Amazon and the coast may be due to their recent introduction to Peru, limited internal migration, lack of widespread SP use, control efforts, the Andes Mountains, and/or differences in vector populations. Our interpretation is consistent with our data suggesting recent population expansion of clonet B. We stress that testing for MDE, bottlenecks, and population expansions requires polymorphic markers, which are difficult to locate in clonets. Clonet A did not share a similar signature of recent expansion, which might have been due to an earlier introduction to the region, a more diverse founding population, or outbreeding with clonets B and/or C based on shared neutral markers.

Clonet C carried the CVMNT-A allele, lacked highly resistant SP- resistant genotypes, and was only found in Padre Cocha and Pampa Hermosa. Indeed, a previous study using samples from across South America only reported this allele in Padre Cocha [32]. Furthermore, a CVMNT allele that grouped with SVMNT was reported among 12 samples collected in Iquitos and two samples from Tabatinga, Brazil, which neighbors Caballococha [15], suggesting clonet C may have also been found in the Amazon basin bordering Peru. Clonet C carried mostly SP sensitive *dhfr* and *dhps* genotypes, which may share ancestry with the alleles found in clonet A and B. Therefore, it is reasonable to argue that clonet C may represent a vestige of an ancestral Amazonia lineage which has been present in Peru for a sufficient period to allow recombination with other clonets. It may be a remnant of the CQ-resistant lineage hypothesized to have developed in southern Rondônia. This would imply it entered eastern Peru from Brazil sometime after the development of CQ resistance in Rondônia in 1960, but prior to the development of SP resistance (Figure 7).

Clonets D and E had divergent neutral backgrounds and were associated with the CVMNT-B allele, the *pfmdr1* β lineage, but not the highly resistant SP genotypes. Based on these commonalities between the clonets D and E, we propose that these two clonets may have spread from the Colombia to Ecuador and then Peru (Figure 7). Clonet D carried a unique 108-C *dhfr* haplotype and a unique synonymous 540 *dhps* mutation not reported elsewhere in South America, potentially indicating genetic drift. Clonet D may represent the CQ-resistant coastal lineage that was argued to have spread from the coast of Ecuador between 1976 and 1980 [53]. It may have spread from Padre Cocha and Caballococha into Ullpayacu or from a bordering Ecuadorian site [48]. Our findings suggest that clonet C and D may have been in Peruvian Amazon longer than clonet A or B, though the recent history of clonet E is ambiguous.

Clonet E was likely introduced to coastal Peru from a more direct coastal migration sometime after 1976 and recently spread into the Peruvian Western Amazon. Clonet E had the least microsatellite variation of the clonets and our analysis suggests this clonet has undergone a bottleneck. However, there was a rapid increase in malaria cases in the region during the 1990s and our study suggests that only clonet E was present. The lack of a statistically significant population expansion might have been caused by more than one clonet E-like lineage invading Peru since 1976 or the sheer lack of genetic diversity overwhelming the statistical test. However, there was a rapid increase in malaria cases in the region during the 1990s and our study suggests that only clonet E was present.

Our findings suggest that the two *pfmdr1* lineages, α and β, have different geographical distributions. Based on our data we predict the α haplotype (found in clonets A, B and a major subset of C, and a few from D) evolved in the Amazon interior of South America while the β *pfmdr1* haplotype (clonets D, E, and to a lesser extent C) evolved on the Pacific Coast or the nearby western Amazon interior. The breakdown in this pattern in clonet C and D is presumably due to outcrossing. This is consistent with a study of *pfmdr1* from Colombia, Brazil, and Guyana, which found that *pfmdr1* haplotypes from Colombia and Guyana were quite distinct [12].

Clonet breakdown in Iquitos (2006–2007) indicates recombination between four different clonets multiple times over the preceding 7–8 years due to increased transmission. There were only a small number of original clonets seen in this region in 1999 (clonets A,B,C, and D) that persisted until 2006–2007 (Table 3). The remaining isolates appeared to be recombinants of clonets B and C, C and D, or, in a few isolates, recombinants of clonets B, C, and D or A, B, and C. We suggest the frequencies of these recombinants are at least partly influenced by the replacement of SP with ACT in this region in 2001. In support of this argument, the SP resistant clonets A and B rapidly declined by 2006–2007 and we showed earlier that the SP resistant genotypes they carried had significantly declined during this period [26], [54]. But beyond this, there was a distinct absence of clonet hybrid offspring which carried SP resistant alleles in 2006–2007 (Table 3), even though parasites carrying these alleles had been prevalent in 1999. If such sexual recombination was just as likely to have occurred, the marked decline of offspring with SP resistant profiles suggests they had lower fitness after SP was removed. These observations illustrate that the parasite clonal lineages underwent rapid changes in population structure due to the increase in malaria, which allowed for outbreeding at the same time that new drug policies altered selection pressure.

Given the large scale migrations within the Amazon basin, periodic epidemics [55], and changes in drug policy there may have been opportunities for outcrossing to occur outside of Peru along with selection pressure from various drugs. Yet it appears that SP resistance may be fixed in the remaining Amazon basin [7] and therefore, multiresistant parasites are likely to persist. This is true in Venezuela, where *pfcrt*, *pfmdr1*, *dhfr*, and *dhps* were linked [9] and other parasites were in linkage for various antigenic genes [13], [56], [57]. However, SP sensitive parasites could disperse into the greater Amazon from Peru due to the development of the Interoceanic Highway, which will connect Atlantic ports of Brazil with Pacific ports of Peru. As countries in the region have moved away from SP use, these SP sensitive parasites may outcompete the already established resistant parasites. Indeed, such migration may have already occurred based on the presence of SP sensitive *dhfr* and *dhps* alleles in the Colombian Amazon [58]. In summary, our study suggests that the molecular characterization of population structure and drug resistance profiles of *P. falciparum* will provide valuable insight into how control programs influence the underlying dynamics and evolution of parasites. Such studies may help to predict the genetic profiles (eg: drug resistant profile) of vestigial parasite populations during and after malaria elimination programs and predict the genetic profiles of parasites that may reappear in subsequent outbreaks.

## References

[1] Ruebush T, Neyra D, Cabezas C (2004). Modifying national malaria treatment policies in Peru.. Journal of Public Health Policy.

[2] Colbourne M (1962). Prospects for malaria eradication:: With special reference to the Western Pacific.. Transactions of the Royal Society of Tropical Medicine and Hygiene.

[3] Gabaldon A (1983). Malaria eradication in Venezuela: doctrine, practice, and achievements after twenty years.. The American journal of tropical medicine and hygiene.

[4] Anderson TJC, Haubold B, Williams JT, Estrada-Francos JG, Richardson L (2000). Microsatellite markers reveal a spectrum of population structures in the malaria parasite *Plasmodium falciparum*.. Molecular Biology and Evolution.

[5] Albrecht L, Castineiras C, Carvalho B, Ladeia-Andrade S, Santos da Silva N (2010). The South American *Plasmodium falciparum var* gene repertoire is limited, highly shared and possibly lacks several antigenic types.. Gene.

[6] Ariey F, Chalvet W, Hommel D, Peneau C, Hulin A (1999). *Plasmodium falciparum* parasites in French Guiana: limited genetic diversity and high selfing rate.. The American Journal of Tropical Medicine and Hygiene.

[7] Cortese JF, Caraballo A, Contreras CE, Plowe CV (2002). Origin and dissemination of *Plasmodium falciparum* drug-resistance mutations in South America.. Journal of Infectious Diseases.

[8] Ferreira M, Liu Q, Kaneko O, Kimura M, Tanabe K (1998). Allelic diversity at the merozoite surface protein-1 locus of *Plasmodium falciparum* in clinical isolates from the southwestern Brazilian Amazon.. The American Journal of Tropical Medicine and Hygiene.

[9] Griffing S, Syphard L, Sridaran S, McCollum A, Mixson-Hayden T (2010). *Pfmdr1* amplification and fixation of chloroquine resistant *pfcrt* alleles in Venezuela.. Antimicrobial Agents and Chemotherapy.

[10] Machado RLD, Povoa MM, Calvosa VSP, Ferreira MU, Rossit ARB (2004). Genetic structure of *Plasmodium falciparum* populations in the Brazilian Amazon region.. Journal of Infectious Diseases.

[11] McCollum AM, Mueller K, Villegas L, Udhayakumar V, Escalante AA (2007). Common origin and fixation of *Plasmodium falciparum dhfr* and *dhps* mutations associated with sulfadoxine-pyrimethamine resistance in a low-transmission area in South America.. Antimicrobial Agents and Chemotherapy.

[12] Mehlotra RK, Mattera G, Bockarie MJ, Maguire JD, Baird JK (2008). Discordant patterns of genetic variation at two chloroquine resistance loci in worldwide populations of the malaria parasite *Plasmodium falciparum*.. Antimicrobial Agents and Chemotherapy.

[13] Tami A, Ord R, Targett G, Sutherland C (2003). Sympatric *Plasmodium falciparum* isolates from Venezuela have structured var gene repertoires.. Malaria Journal.

[14] Urdaneta L, Plowe C, Goldman I, Lal AA (1999). Point mutations in dihydrofolate reductase and dihydropteroate synthase genes of *Plasmodium falciparum* isolates from Venezuela.. The American Journal of Tropical Medicine and Hygiene.

[15] Vieira PP, Ferreira MU, Alecrim MG, Alecrim WD, Silva LHP (2004). *Pfcrt* polymorphism and the spread of chloroquine resistance in *Plasmodium falciparum* populations across the Amazon Basin.. Journal of Infectious Diseases.

[16] Tibayrenc M, Ayala FJ (1991). Towards a population genetics of microorganisms: the clonal theory of parasitic protozoa.. Parasitology Today.

[17] Tibayrenc M, Ayala FJ (2002). The clonal theory of parasitic protozoa: 12 years on.. Trends in Parasitology.

[18] Organización Mundial de la Salud-OMS (1991). Situacion de los programas de malaria en las americas. XXXIX informe.

[19] Chowell G, Munayco C, Escalante A, McKenzie F (2009). The spatial and temporal patterns of falciparum and vivax malaria in Perú: 1994–2006.. Malaria Journal.

[20] Marquino W, Ylquimiche L, Hermenegildo Y, Palacios A, Falconi E (2005). Efficacy and tolerability of artesunate plus sulfadoxine-pyrimethamine and sulfadoxine-pyrimethamine alone for the treatment of uncomplicated *Plasmodium falciparum* malaria in Peru.. The American Journal of Tropical Medicine and Hygiene.

[21] Aramburu GJ, Ramal AC, Witzig R (1999). Malaria reemergence in the Peruvian Amazon region.. Emerging Infectious Diseases.

[22] Durand S, Marquino W, Cabezas C, Utz G, Fiestas V (2007). Unusual pattern of *Plasmodium falciparum* drug resistance in the northwestern Peruvian Amazon region.. The American Journal of Tropical Medicine and Hygiene.

[23] Huaman MC, Roncal N, Nakazawa S, Long T, Gerena L (2004). Polymorphism of the *Plasmodium falciparum* multidrug resistance and chloroquine resistance transporter genes and in vitro susceptibility to aminoquinolines in isolates from the Peruvian Amazon.. The American Journal of Tropical Medicine and Hygiene.

[24] Magill A, Zegarra J, Garcia C, Marquiño W, Ruebush T (2004). Efficacy of sulfadoxine-pyrimethamine and mefloquine for the treatment of uncomplicated *Plasmodium falciparum* malaria in the Amazon basin of Peru.. Revista da Sociedade Brasileira de Medicina Tropical.

[25] Marquino W, MacArthur J, Barat L, Oblitas F, Arrunategui M (2003). Efficacy of chloroquine, sulfadoxine-pyrimethamine, and mefloquine for the treatment of uncomplicated *Plasmodium falciparum* malaria on the north coast of Peru.. The American journal of tropical medicine and hygiene.

[26] Bacon DJ, McCollum AM, Griffing SM, Salas C, Soberon V (2009). Dynamics of malaria drug resistance patterns in the Amazon basin region following changes in Peruvian national treatment policy for uncomplicated malaria.. Antimicrobial Agents and Chemotherapy.

[27] Uhlemann A, Yuthavong Y, Fidock D, Sherman I, Sherman I (2005). Mechanisms of antimalarial drug action and resistance.. Molecular Approaches to Malaria.

[28] Roper M, Torres R, Goicochea C, Andersen E, Guarda J (2000). The epidemiology of malaria in an epidemic area of the Peruvian Amazon.. The American Journal of Tropical Medicine and Hygiene.

[29] Vinayak S, Alam MT, Mixson-Hayden T, McCollum AM, Sem R (2010). Origin and evolution of sulfadoxine resistant *Plasmodium falciparum*.. PLoS Pathogens.

[30] Nair S, Nash D, Sudimack D, Jaidee A, Barends M (2007). Recurrent gene amplification and soft selective sweeps during evolution of multidrug resistance in malaria parasites.. Molecular Biology and Evolution.

[31] Nash D, Nair S, Mayxay M, Newton PN, Guthmann JP (2005). Selection strength and hitchhiking around two anti-malarial resistance genes.. Proceedings of the Royal Society of London Series B: Biological Sciences.

[32] Wootton JC, Feng X, Ferdig MT, Cooper RA, Mu J (2002). Genetic diversity and chloroquine selective sweeps in *Plasmodium falciparum*.. Nature.

[33] Anderson T, Su X, Bockarie M, Lagog M, Day K (1999). Twelve microsatellite markers for characterization of *Plasmodium falciparum* from finger-prick blood samples.. Parasitology.

[34] Excoffier L, Laval G, Schneider S (2005). Arlequin (version 3.0): an integrated software package for population genetics data analysis.. Evolutionary Bioinformatics Online.

[35] Slatkin M (1995). A measure of population subdivision based on microsatellite allele frequencies.. Genetics.

[36] Raymond M, Rousset F (1995). An exact test for population differentiation.. Evolution.

[37] Sankoh AJ, Huque MF, Dubey SD (1997). Some comments on frequently used multiple endpoint adjustment methods in clinical trials.. Statistics in Medicine.

[38] Cornuet J, Luikart G (1996). Description and power analysis of two tests for detecting recent population bottlenecks from allele frequency data.. Genetics.

[39] Bandelt H, Forster P, Rohl A (1999). Median-joining networks for inferring intraspecific phylogenies.. Molecular Biology and Evolution.

[40] Annan Z, Durand P, Ayala F, Arnathau C, Awono-Ambene P (2007). Population genetic structure of *Plasmodium falciparum* in the two main African vectors, *Anopheles gambiae* and *Anopheles funestus*.. Proceedings of the National Academy of Sciences.

[41] Conway DJ, Roper C, Oduola AMJ, Arnot DE, Kremsner PG (1999). High recombination rate in natural populations of *Plasmodium falciparum*.. Proceedings of the National Academy of Sciences.

[42] Escalante A, Cornejo O, Rojas A, Udhayakumar V, Lal A (2004). Assessing the effect of natural selection in malaria parasites.. Trends in Parasitology.

[43] Garcia-Martins G (1972). Status of malaria eradication in the Americas.. The American Journal of Tropical Medicine and Hygiene.

[44] Wright S (1943). Isolation by distance.. Genetics.

[45] Wright S (1940). Breeding structure of populations in relation to speciation.. American Naturalist.

[46] Branch OLH, Sutton PL, Castro JC, Barnes C, Hussin J (2011). Plasmodium Falciparum Genetic Diversity Maintained and Amplified Over Five Years of a Low Transmission Endemic in the Peruvian Amazon.. Molecular Biology and Evolution.

[47] Dharia N, Plouffe D, Bopp S, González-Páez G, Lucas C (2010). Genome-scanning of Amazonian *Plasmodium falciparum* shows subtelomeric instability and clindamycin-resistant parasites.. Genome Research.

[48] Chauca H, Quintana J (1993). Evaluación in vivo de la respuesta de *Plasmodium falciparum* a la cloroquina en foco carretera Yurimaguas-Tarapoto (Región Loreto).. Revista Peruana de Epidemiología.

[49] World Health Organization (1985). World malaria situation 1983.. World Health Statistics Quarterly.

[50] Clyde DF (1987). Genesis of Chloroquine-Resistant *Plasmodium falciparum* in the America Region.. La Medicina Tropicale Nella Cooperazione Allo Sviluppo.

[51] Moore DV, Lanier JE (1961). Observations on two *Plasmodium falciparum* infections with an abnormal response to chloroquine.. The American Journal of Tropical Medicine and Hygiene.

[52] Souza JM (1992). Epidemiological distribution of Plasmodium falciparum drug resistance in Brazil and its relevance to the treatment and control of malaria.. Memórias do Instituto Oswaldo Cruz, Rio de Janeiro.

[53] Payne D (1987). Spread of chloroquine resistance in *Plasmodium falciparum*.. Parasitology Today.

[54] Zhou Z, Griffing SM, de Oliveira AM, McCollum AM, Quezada WM (2008). Decline in sulfadoxine-pyrimethamine-resistant alleles after change in drug policy in the Amazon region of Peru.. Antimicrobial Agents and Chemotherapy.

[55] Marques C (1987). Human migration and the spread of malaria in Brazil.. Parasitology Today.

[56] Tami A, Grundmann H, Sutherland C, McBride J, Cavanagh D (2002). Restricted genetic and antigenic diversity of *Plasmodium falciparum* under mesoendemic transmission in the Venezuelan Amazon.. Parasitology.

[57] Urdaneta L, Lal A, Barnabe C, Oury B, Goldman I (2001). Evidence for clonal propagation in natural isolates of *Plasmodium falciparum* from Venezuela.. Proceedings of the National Academy of Sciences of the United States of America.

[58] Corredor V, Murillo C, Echeverry DF, Benavides J, Pearce RJ (2010). Origin and dissemination across the Colombian Andes Mountains range of sulfadoxine-pyrimethamine resistance in *Plasmodium falciparum*.. Antimicrobial Agents and Chemotherapy.

